# Effect of the Processing on the Resistance–Strain Response of Multiwalled Carbon Nanotube/Natural Rubber Composites for Use in Large Deformation Sensors

**DOI:** 10.3390/nano11071845

**Published:** 2021-07-16

**Authors:** Xingyao Liu, Rongxin Guo, Rui Li, Hui Liu, Zhengming Fan, Yang Yang, Zhiwei Lin

**Affiliations:** Key Laboratory of Disaster Reduction in Civil Engineering, Faculty of Civil Engineering and Mechanics, Kunming University of Science and Technology, Kunming 650500, China; xingyaoliu@stu.kust.edu.cn (X.L.); liruiking@kust.edu.cn (R.L.); 15079743713@163.com (H.L.); 20202210032@stu.kust.edu.cn (Z.F.); yangyang0416@kust.edu.cn (Y.Y.); lzw@kust.edu.cn (Z.L.)

**Keywords:** resistance–strain response, carbon nanotube, natural rubber composites, processing method, deformation monitoring

## Abstract

The dispersion, electrical conductivities, mechanical properties and resistance–strain response behaviors of multiwalled carbon nanotube (MWCNT)/natural rubber (NR) composites synthesized by the different processing conditions are systematically investigated at both macro- and micro-perspectives. Compared with the solution and flocculation methods, the two roll method produced the best MWCNTs distribution since the materials are mixed by strong shear stress between the two rolls. An excellent segregated conductive network is formed and that a low percolation threshold is obtained (~1 wt.%) by the two roll method. Different from the higher increases in conductivity for the composites obtained by the solution and flocculation methods when the MWCNT content is higher than 3 wt.%, the composite prepared by the two roll method displays obvious improvements in its mechanical properties. In addition, the two roll method promotes good stability, repeatability, and durability along with an ultrahigh sensitivity (*GF*_max_ = 974.2) and a large strain range (*ε* = 109%). The ‘shoulder peak’ phenomenon has not been observed in the composite prepared by the two roll method, confirming its potential for application as a large deformation monitoring sensor. Moreover, a mathematical model is proposed to explain the resistance–strain sensing mechanism.

## 1. Introduction

Elastomer nanocomposites (ENCs), due to their excellent strain sensing performance, outstanding flexibility and good reproducibility, have attracted increasing attention in many fields, including aerospace devices [1,2], body movement monitoring [3,4], electronic skin devices [5,6], electromagnetic shielding devices [7,8], gas barriers [9,10], tire enhancement [11,12] and structural health monitoring [13,14]. ENCs are usually fabricated by incorporating excellent nanofillers into the elastomer matrix. In fact, the performance of ENCs is significantly dependent on the selections of conductive nanofillers and matrices. Carbon nanotubes (CNTs) are favorable nanofillers due to their relatively low cost, low density, large aspect ratio, excellent mechanical, electrical and thermal conductivity properties [15,16,17,18], thus, CNTs have become the most potential carbon materials, especially MWCNTs [19]. Natural rubber, as a typical nonpolar macromolecular polymer matrix material, has been widely used as an ENCs matrix in engineering due to its good viscoelasticity, large deformability, electrical insulation, heat insulation, waterproofness and plasticity [20,21,22,23,24].

Many processing methods including emulsion mixing, solution blending and flocculation method are known for the manufacture of rubber composites. For instance, Renato Torres and coauthors [5] used the emulsion mixing method to prepare MWCNT/NR sensing materials with a percolation threshold of 4%. At 50% strain, the gauge factor was 3.6 ± 0.2, indicating that the prepared composite material had a certain repeatability and linearity. Neena George et al. [9] prepared MWCNT/NR composites by ultrasonic-assisted dispersion solution blending of acidified latex and obtained composites with separation networks. The prepared composite had good dispersion and a low percolation threshold. Compared with the unfilled NR, the tensile strength increased by 61%, the modulus increased by 75%, and the shear strength increased by 59% for 0.5 parts per hundred rubber (phr) MWCNT/NR. Kim et al. [25] fabricated the styrene butadiene rubber composite by flocculation method. The percolation threshold for the electrical conductivity was achieved at a low filler concentration (0.5 wt.%). However, emulsion mixing, solution blending, and flocculation method are limited to laboratory efforts due to their low potential for scalability, high processing time duration and low-cost efficiency, even some organic solvents will be consumed during the preparation. Besides the above processing methods, the two roll method with huge potential for cost-effectiveness and scalability has been introduced to produce the rubber composite. Such as N. Tamil Selvan [26] prepared MWCNT/NR composites with a percolation threshold of 2.5 phr and a *t* value of 2.31 by the two roll method. The effects of filler type, filler concentration, plasticizer dose and crosslinking density of the main rubber on the strain sensing properties were analyzed. However, the two roll method suffers from the poor dispersion of additives. A high concentration of nanofillers is required to produce enough change in electric properties for ENCs.

As mentioned above, several processing methods have been widely used in the preparation of composite, but, up until now, the effects of the preparation process on the dispersion, microstructure morphology, mechanical properties, dynamic resistance–strain response and interfacial strength of MWCNT/NR composites have not been systematically researched. In addition, as reported in the literature [27] and shown in Table S1, achieving a good combination between exhibiting a broad detection range and high sensitivity is still challenging. However, to overcome the shortcoming of the preparation methods and produce the composite with a wide sensing range and high sensitivity. It is essential to clarify and master the effect of the various preparation methods on the conductive network characteristics, conductive sensitivity mechanism and interfacial interaction as well as the reinforcement mechanism of the composites for the preparation of advanced composites, all of which are the basis for application in all fields.

Herein, considering that the properties of the composites are mainly affected by the dispersion state of the filler in the matrix, three different and representative fabrication methods in the field of nanocomposite sensors (solution method, flocculation method and two roll method) are applied and conducted to prepare MWCNT/NR composite. The influence of the processing methods on the dispersion, mechanical properties, electrical conductivity, resistance–strain response and microstructure morphology of the MWCNT/NR composites is systematically investigated. The interfacial interaction and reinforcement mechanism of the composites are discussed and clarified by microstructural analysis, Fourier transform infrared (FTIR) spectroscopy and Raman spectroscopy. Finally, a mathematical model is developed to explore the resistance–strain response mechanism of the different processing methods.

## 2. Experiment

### 2.1. Raw Materials

Natural rubber latex (NRL) with 60 wt.% solid content was purchased from Zhengmao Petrochemical Co., Ltd., Maoming City, Guangdong, China. Multiwalled carbon nanotubes (MWCNTs) with lengths of 10–20 µm, outer diameters of 4–6 nm, as shown in Figure S1, specific surface areas of 500–700 m^2^/g and a purity of >98% were purchased from Chengdu Organic Chemicals Co., Ltd., Chinese Academy of Sciences, Chengdu, China. Other agents including tetrahydrofuran (THF), hydrochloric acid (HCl), deionized water, dicumyl peroxide (DCP) and cetyl trimethyl ammonium bromide (CTAB) were obtained from Kunming Kerui Instrument Co., Ltd., Kunming, China. All of the above are all commercially available and without any extra treatment.

### 2.2. Preparation Methods

**Figure 1:**
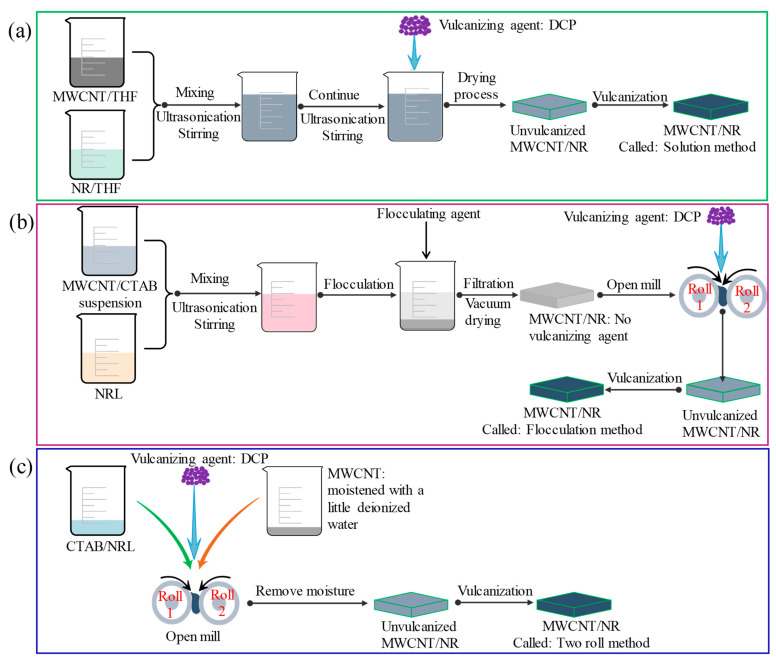
Schematic illustration for preparing MWCNT/NR composites: (**a**) solution method; (**b**) flocculation method; (**c**) two roll method. (MWCNT: multiwalled carbon nanotube; NR: natural rubber; NRL: natural rubber latex; THF: tetrahydrofuran; DCP: dicumyl peroxide; CTAB: cetyl trimethyl ammonium bromide).

(1) Solution method (Figure 1a)

MWCNT/THF and NRL/THF dispersions were obtained through ultrasonic dispersion with stirring for 1 h at room temperature. The two dispersions were mixed and continuous ultrasonic with stirring was performed for 0.75 h, then DCP curing agent was added, and ultrasonic with stirring was performed for 0.25 h to obtain a black mixture at 40 °C. The black mixture was transferred to a drying oven to remove the THF and moisture. Finally, the MWCNT/natural rubber (NR) composites were obtained by vulcanizing at 155 ± 5 °C, 10 MPa for 10 min. The MWCNT/NR composite was named as “solution method” for the sake of a concise and clear description.

(2) Flocculation method (Figure 1b)

To improve the flocculation efficiency of the disperse suspension of MWCNTs and NR and the dispersion of MWCNTs in NR, CTAB was used in the flocculation method. MWCNTs and CTAB were mixed and dispersed in deionized water according to mass ratio 1:1 for 1 h. Then mix and disperse NRL and the MWCNT/CTAB dispersion for 2 h, and then HCl was added for flocculation to obtain the stable dispersion state of MWCNT/NR mixture. The mixture was repeatedly washed and soaked in deionized water for not less than 5 h to remove the hydrochloric acid solution and dried at 60 °C in a vacuum oven for 24 h, and then a two roll mill was used for the mixing of the vulcanizing agent DCP. The rolls were set to a temperature of approximately 35 °C, a speed of 25 rpm with a friction ratio of 1.2:1, and a nip gap of 0.5 mm. A total mixing time of 10 min was used for the dispersion of DCP into MWCNT/NR mixture. Finally, the MWCNT/NR composite was obtained by the same curing conditions as the solution method, which is named as “flocculation method”. To analyze the effect of CTAB on composites, 6 wt.% CTAB/NR was prepared using the same method described above.

(3) Two roll method (Figure 1c)

CTAB and NRL were mixed and stirred to demulsify. MWCNTs were moistened with a little deionized water to avoid dry MWCNTs flutter and mixed with NRL/CTAB on a two roll mill at 35 ± 5 °C. The rolls were first set to a speed of 25 rpm with a friction ratio of 1.2:1, and a nip gap of 1.5 mm. After 5 min initial mixing, the nip gap is adjusted to 0.5 mm to further promote the dispersion of MWCNTs into NR for 5 min. Subsequently, the DCP was added into the dispersed materials and further mixed for 15 min. Finally, the MWCNT/NR composite was obtained by the same curing conditions as the solution method, which is named as “two roll method”. Comparing with the other two methods, the two roll method provides better dispersion of MWCNTs since the strong shear force between two rolls provides sufficient mechanical mixing for the materials.

## 3. Results and Discussion

### 3.1. Influence of the Processing Method on the Dispersion of MWCNTs in the NR Matrix

**Figure 2:**
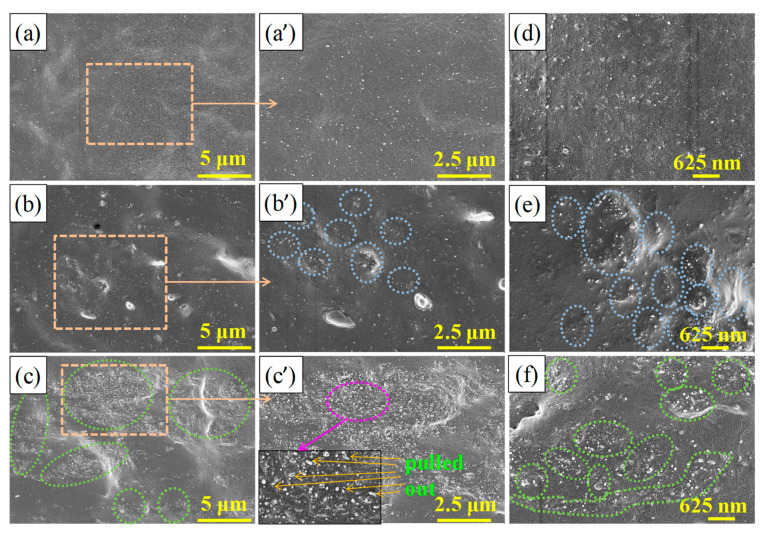
FESEM images of the MWCNT distribution in the cross-section of the 6 wt.% MWCNT/NR composites produced with different processing methods: two roll method (**a**,**a’**,**d**), flocculation method (**b**,**b’**,**e**), and solution method (**c**,**c’**,**f**,**a’**,**b’**,**c’**) are partial enlargement of (**a**–**c**). (FESEM: field emission scanning electron microscopy; MWCNT: multiwalled carbon nanotube; the blue circle and green circle represent the MWCNT clusters in the flocculation and the two roll method, respectively).

The dispersion of MWCNTs in the NR matrix is a key factor in determining the performance of MWCNT/NR composites [28]. Figure 2 shows the dispersion levels in the cross-section (field emission scanning electron microscopy (FESEM) observation) of the 6 wt.% MWCNT/NR composites prepared by the three different processing methods. As shown in Figure 2a,a’,d, MWCNTs are homogeneously dispersed with few small clusters observed in the NR matrix by the shear stress caused by the two roll mill [28]. Regarding the solution method, more and larger MWCNTs clusters are observed (highlighted by the green circles in Figure 2c,f), which play a significant role in improving the volume conductivity of composites when the clusters are connected or reach tunneling conditions. After using the flocculation method, some MWCNTs cluster to form island-like areas (dotted circle in Figure 2b’,e) that are connected or close to each other, thereby forming a conductive path that is highlighted by the blue circle in Figure 2b’,e. Compared with the solution method, the clusters of MWCNTs with the flocculation method are fewer and smaller. However, the more numerous and larger clusters are uniformly dispersed in the NR matrix for the flocculation and solution method compared with the two roll method.

**Figure 3:**
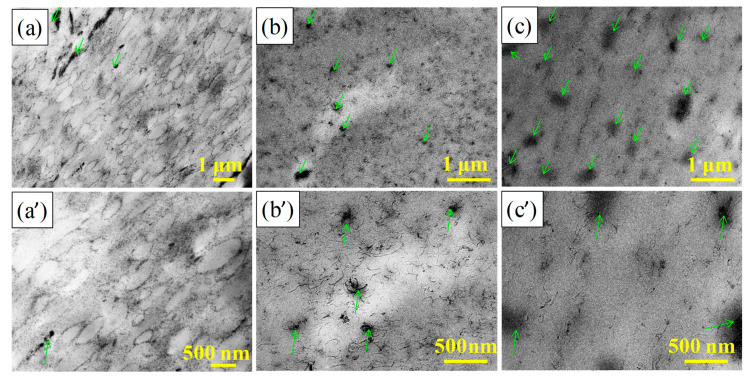
TEM images of the 4 wt.% MWCNT/NR composites produced with different processing methods: two roll method (**a**,**a’**), flocculation method (**b**,**b’**), and solution method (**c**,**c’**). (TEM: transmission electron microscopy; MWCNT: multiwalled carbon nanotube; the green arrow represents the MWCNT clusters in the MWCNT/NR composite).

Figure 3 shows the transmission electron microscopy (TEM) topography of the 4 wt.% MWCNT/NR composites prepared by the three procedures. It is clearly noted that the MWCNTs are aligned randomly and dispersed uniformly in the NR matrix for the flocculation and solution method (Figure 3b,b’,c,c’). Meanwhile, the serious clusters are observed easily (the green arrow in Figure 3b,b’,c,c’), especially the solution method. However, a more integrated and segregated network is produced along the edges of NR regions when the two roll method is employed (Figure 3a,a’), which means MWCNTs are dispersed well by the two roll method.

**Figure 4:**
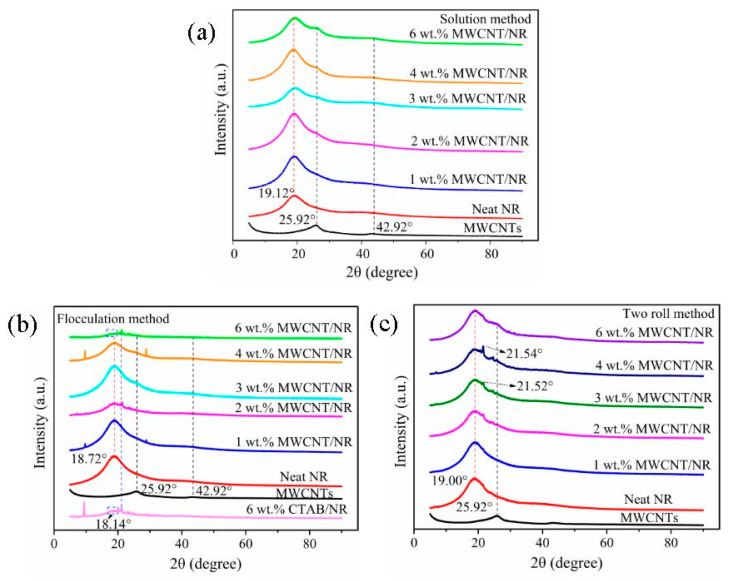
XRD patterns of the MWCNT/NR composites prepared by (**a**) the solution method, (**b**) flocculation method, and (**c**) two roll method. (XRD: X-ray diffraction; MWCNT: multiwalled carbon nanotube; NR: natural rubber; CTAB: cetyl trimethyl ammonium bromide).

XRD technique can be used to assess the stacking of MWCNTs by comparing scattering patterns of the composites with those of neat NR. Figure 4 shows XRD spectra of MWCNTs, neat NR and MWCNT/NR composites at different MWCNT content for the three processing methods. When the loading exceeds 2 wt.% (solution method) and 3 wt.% (flocculation method), the appearance of the characteristic diffraction peak of MWCNTs (Figure S2a) demonstrates that the MWCNTs have obvious stacking in the NR matrix when the solution method and flocculation method are used (as shown in Figure 4a,b), especially at a high loading of 6 wt.% MWCNT. Regarding the MWCNT/NR composite from the flocculation method, the attenuation of the NR peak may be related to the addition of cetyltrimethylammonium bromide (CTAB) (as shown in Figure S2b,c). Different from the other two methods, there is no obvious characteristic peak of MWCNTs except with 6 wt.% MWCNT/NR composite prepared by two roll method (Figure 4c), which is attributed to the strong shear stress caused by the two roll method effectively eliminates stacked MWCNTs [29]. A small sharp CTAB characteristic peak appears near 2θ = 21.50° due to the CTAB added.

### 3.2. Conductivity

**Figure 5:**
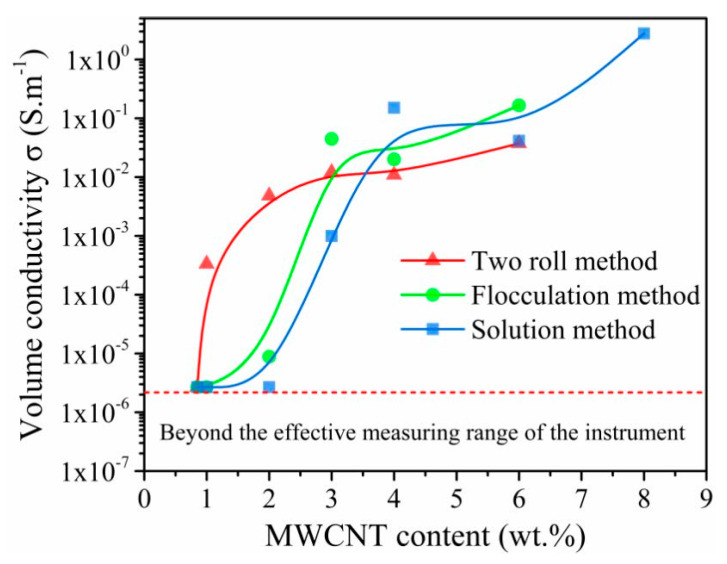
Volume conductivities of the MWCNT/NR composites as a function of their MWCNT content. (MWCNT: multiwalled carbon nanotube; NR: natural rubber).

The volume conductivity of the composite depends on the dispersion of fillers and the conductive network structure in the rubber matrix [30], and the calculation method is shown in the Supplementary Material. Figure 5 shows the relationship of the volume conductivity via MWCNT contents with the three processing methods, and the unstrained volume conductivity increased with an increase of MWCNT content, exhibiting a typical percolation behavior. In this work, only the conductivity of MWCNT/NR with a conductivity above 2 × 10^−6^ S·m^−1^ was tested and analyzed due to the limitation of the instrument. Clearly, the percolation thresholds for composites with the two roll method, flocculation method and solution method are ~1 wt.%, ~2 wt.% and ~3 wt.%, respectively. Moreover, the two roll method exhibits a higher volume conductivity below the threshold content 3 wt.%, which is related to the formation of the segregated conductive networks. The segregated conductive network has been reported to be the most promising strategy to significantly decrease the percolation threshold and the dense conductive paths at low filler loading [31]. However, the conductivity of composite from the two roll method is lower than that of the composite prepared by the other two methods when the MWCNT content exceeds the threshold. These results suggest that a complete conductive network for composites prepared by the solution method and flocculation method is formed at high MWCNT contents, which is interpreted as some large agglomerates of MWCNTs are formed, and the construction of a conductive network requires a high MWCNT content [30]. However, there are more effective conductive paths induced by the flocculation method and solution method than by the two roll method when the MWCNT content exceeds the 3 wt.%, thus, the composites from the flocculation method and solution method have higher volume conductivities.

### 3.3. Mechanical Properties

**Figure 6:**
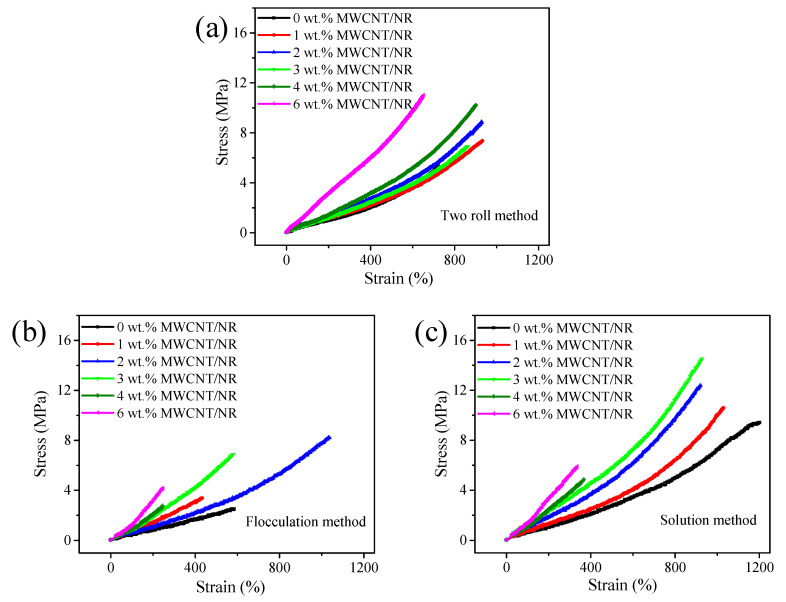
The stress–strain curves of MWCNT/NR composites prepared by the different processing methods: (**a**) two roll method, (**b**) flocculation method, (**c**) solution method. (MWCNT: multiwalled carbon nanotube; NR: natural rubber).

The stress–strain curves of MWCNT/NR composites prepared by different processing methods are summarized in Figure 6. Basically, the stress increases as the strain and MWCNT content, and the tensile strain decreases as the increases of MWCNT content. Regarding both the solution method (Figure 6c) and flocculation method (Figure 6b), the tensile strain is significantly reduced at 4 wt.% and 6 wt.% of MWCNT content which is attributed to the stress concentration caused by MWCNT stacking and interfacial interactions. It is clearly observed that the strain fluctuates strongly with the increase of the MWCNT contents in Figure 6b, which is caused by the internal holes of the composite prepared by the flocculation method, and will be discussed in a later section. In addition, MWCNTs play an active role to increase the tensile strain of composite prepared by two roll method when MWCNT content is less than 4 wt.%, which is quite different from the other two systems, since the two roll method does not cause MWCNT aggregation and that the stress concentration is weaker than other two processing methods [32,33]. To comparing the mechanical properties of composites prepared by three different methods, the relationships of tensile strength, elongation at break and elastic modulus of the composite with MWCNT contents are shown in Figure S3.

### 3.4. Resistance–Strain Response Behavior

#### 3.4.1. The Strain Sensing Behaviors under Uniaxial Strain

**Figure 7:**
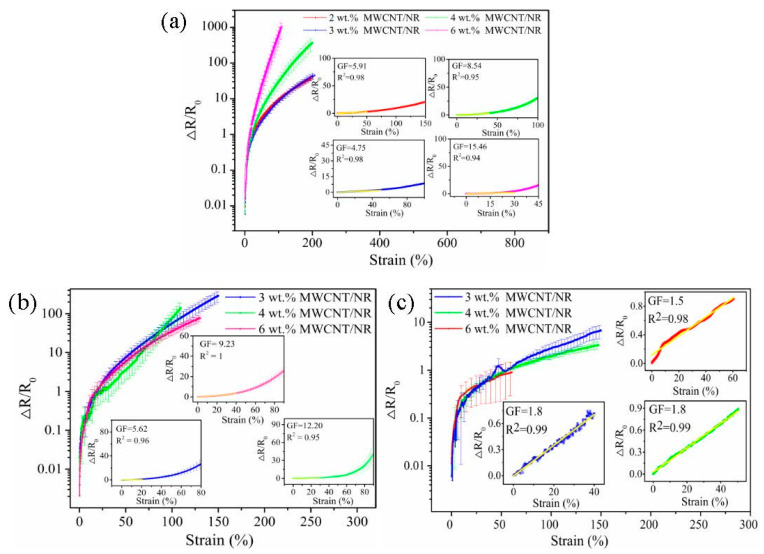
∆*R*/*R*_0_ as a function of the applied strain for MWCNT/NR composites with different MWCNT contents: (**a**) two roll method, (**b**) flocculation method and (**c**) solution method. (∆*R*/*R*_0_: the resistance change fraction; MWCNT: multiwalled carbon nanotube; NR: natural rubber; GF: gauge factor).

The exploration of strain sensing behavior for large deformation sensors is of prime importance. Figure 7 shows the relationship between the resistance change fraction ∆*R*/*R*_0_ (∆*R* = *R* − *R*_0_, where *R* and *R*_0_ are the resistance at applied strain (*ε*) and initial strain (*ε*_0_), respectively) and *ε* of the MWCNT/NR composites from the three processing methods at a rate of 10 mm/min. Clearly, the ∆*R*/*R*_0_ values of the composites increase with an increasing *ε*. This result is induced by the gradual destruction of the conductive network and the steric hindrance affecting electron transfer with an increasing strain, thus leading to increased resistance [34]. It is surprising that the ∆*R*/*R*_0_ of the composite from the two roll method shows a contrary variation trend with strain when compared with that of the composites from the other two systems (Figure 7b,c), and similar trends have been found in the literature [31,35]. Regarding the two roll method, the structural change of the segregated conductive network is greater at a high MWCNT content due to the high segregated conductive network density (Figure 3a), thus, ∆*R*/*R*_0_ is higher at a high MWCNT content than that at a low MWCNT content.

**Figure 8:**
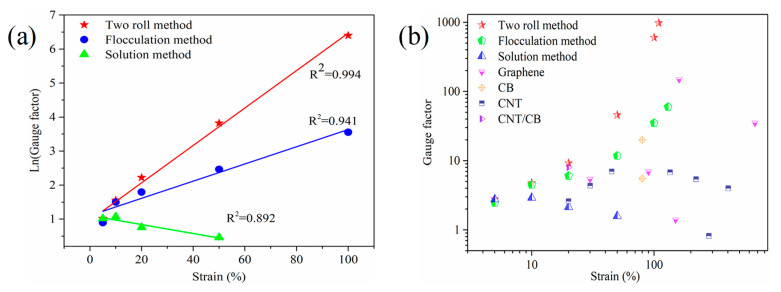
(**a**) Ln(Gauge factor) of 6 wt.% MWCNT/NR from the different processing methods as a function of applied strain; (**b**) summary of the reported literature for flexible strain sensors (see the Table S1 of Supplementary Material for detailed data and references): the maximum value of *GF* plotted versus the strain sensing range. (MWCNT: multiwalled carbon nanotube; NR: natural rubber; CB: carbon black; CNT: carbon nanotubes).

To quantify the resistance–strain response of the MWCNT/NR composites, the gauge factor (*GF*) was calculated, $GF=(\Delta R/R_{0})/\varepsilon$. The insets in Figure 7 shows the *GF* of the composites in the linear region under three conditions. It can be seen that the *GF* values of the two roll method composite are 5.91 (*ε* = 50%, 2 wt.%), 4.75 (*ε* = 45%, 3 wt.%), 8.54 (*ε* = 40%, 4 wt.%) and 15.46 (*ε* = 30%, 6 wt.%). The *GF*_max_ of the flocculation method composite is 12.20 (*ε* = 30%, 4 wt.%). The linear strain range of the solution method composite is >40%, and its *GF*_max_ is only 1.8 (3 wt.% and 4 wt.% MWCNT/NR). From these results, the two roll method and flocculation method have greater sensitivity in the linear region. The 6 wt.% MWCNT/NR from the solution method shows a linear change (coefficient of determination, R^2^ = 0.98) within the sensing range (the inset in Figure 7c), which is caused by its excellent conductivity (Figure 5) and poor deformability (Figure 6) at high loading. Meanwhile, as seen from Figure 8a, the Ln(*GF*) of the composites prepared by the two roll method and flocculation method linearly increase as the applied strain increases (R^2^ > 0.9); in contrast, the Ln(*GF*) shows a decreasing trend for the solution method composite and a lower *GF* (Figure S4), indicating that two roll method composites with the segregated network have a higher *GF* than the other two methods. Compared with the solution method, flocculation method and the other research results (Figure 8b, Table S1), it clearly confirmed that the two roll method has ultrahigh sensitivity and a wide strain range (*ε* = 109%, *GF*_max_ = 974.2), indicating that the two roll method composites have the potential as strain sensors with large deformation and ultrahigh sensitivity.

#### 3.4.2. Dynamic Sensing Behavior

**Figure 9:**
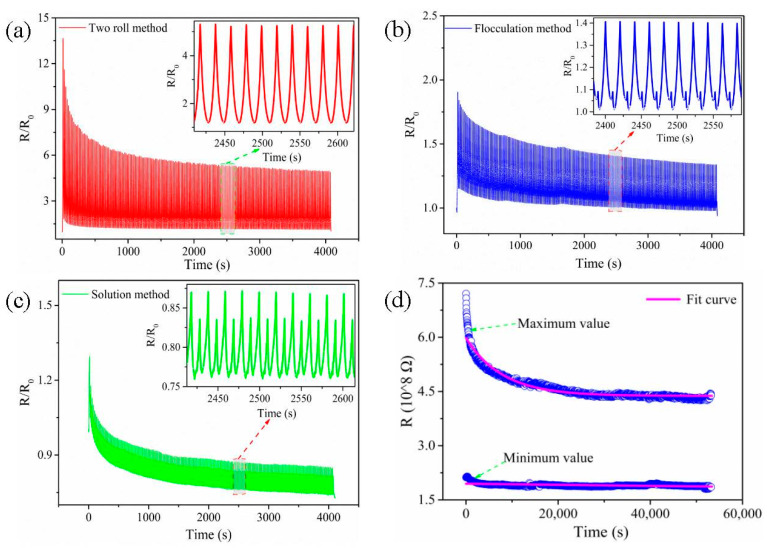
*R*/*R*_0_ under cyclic strain for the 6 wt.% MWCNT/NR composites with a maximum strain of 30% at a strain rate of 50 mm/min: (**a**) two roll method, (**b**) flocculation method, (**c**) solution method and (**d**) the *R* of the two roll method composite for 2400 cycles. (MWCNT: multiwalled carbon nanotube; NR: natural rubber).

To investigate the repeatability, stability and durability of composite in the long-term monitoring, the cyclic loading experiments of 200 loading–unloading cycles with a strain of 30% at a strain rate of 50 mm/min for the 6 wt.% MWCNT/NR composites prepared by the three processing methods are conducted (as shown in Figure 9a–c). It can be seen that the *R*/*R*_0_ values gradually decrease when the cycles increase and tend to be stable finally.

As reported in other studies [27,34,36], the ‘shoulder peak’ phenomenon has also been observed in the solution and flocculation method, especially for the solution method composite (Figure 9c). The existing literature has generally provided a consistent explanation for the origin of the ‘shoulder peak’, both the competition between the destruction and reconstruction of conductive networks during cyclic loading and the viscoelastic properties of the matrix are considered the main reasons [27,37,38]. However, for the two roll method, the ‘shoulder peak’ is not observed during 200 cycles, and excellent monotony of *R*/*R*_0_ variation is shown (Figure 9a). As the promising candidate, it is necessary to further explore the durability of the two roll method composite, 2400 cycles are carried out with a strain of 30% at a loading rate of 50 mm/min (Figure 9d). The maximum and minimum values of the resistance (*R*) are extracted and fitted by MATLAB. The fitting curve shows that the composite from the two roll method has good durability and stability. During the initial cycles, *R*/*R*_0_ undergoes a slight decrease, which is ascribed to the construction of new conductive pathways during the stretching-releasing process. As the number of cycles increases, *R*/*R*_0_ gradually stabilizes because the conductive network achieves an equilibrium state between destruction and reconstruction after a period of self-adjustment, demonstrating the excellent durability of the two roll method composite [39]. In fact, this result is also closely related to the unique segregated conductive network and excellent mechanical properties of the two roll method composite.

**Figure 10:**
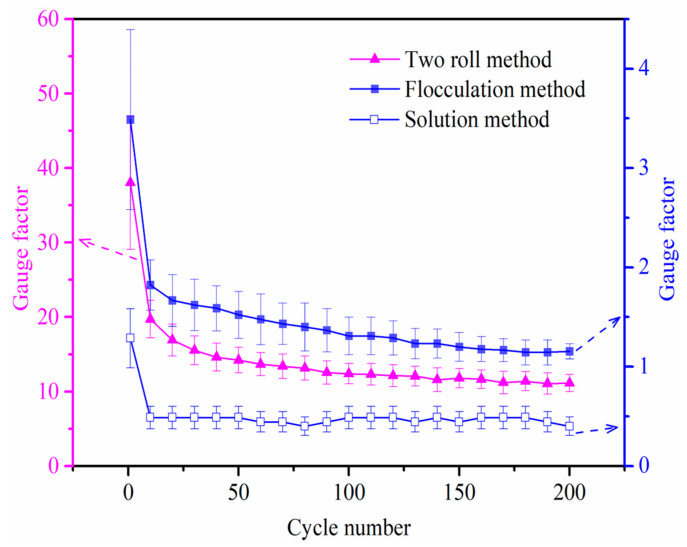
Gauge factors of the composites prepared by three processing methods after various numbers of cycling.

Meanwhile, to investigate the strain sensing properties after several strain cycles, the variation for *GF* value of the composites prepared by three processing methods is shown in Figure 10. It is clearly shown that *GF* decreases dramatically during the initial 20 cycles due to the competition between destruction and reconstruction of the conductive network. Then the decreasing trend of *GF* becomes slow with the increase of cycles, except for the solution method. According to the above testing results, the solution method possesses more MWCNT clusters, high percolation threshold, poor monotony and low *GF*, but a more stable GF is obtained during the cycles after 20 cycles.

### 3.5. Interface and Reinforcement Mechanism

**Figure 11:**
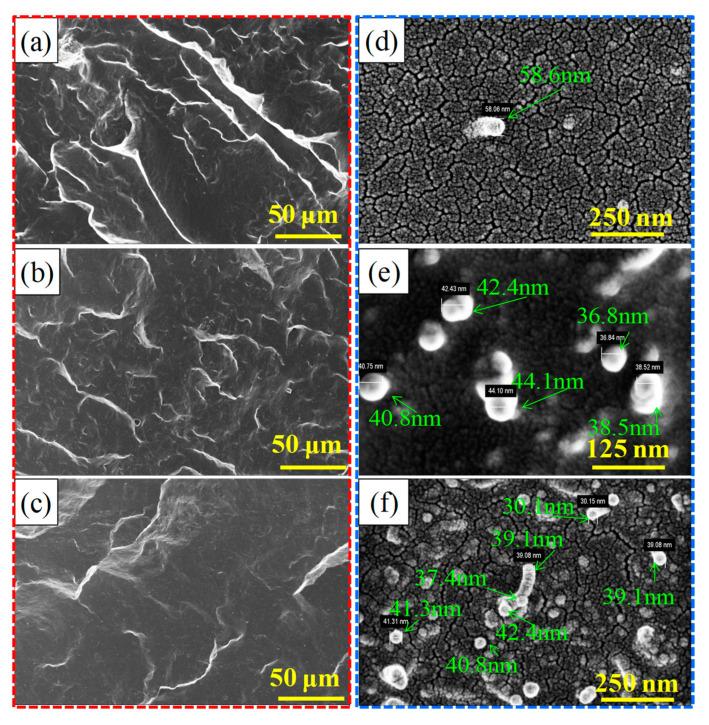
Cross-sectional microtopography (red-dotted rectangle) and pulled-out MWCNTs (blue-dotted rectangle) of the 6 wt.% MWCNT/NR composites from different processing methods: (**a**,**d**) two roll method, (**b**,**e**) flocculation method, and (**c**,**f**) solution method. (MWCNT: multiwalled carbon nanotube; NR: natural rubber).

**Figure 12:**
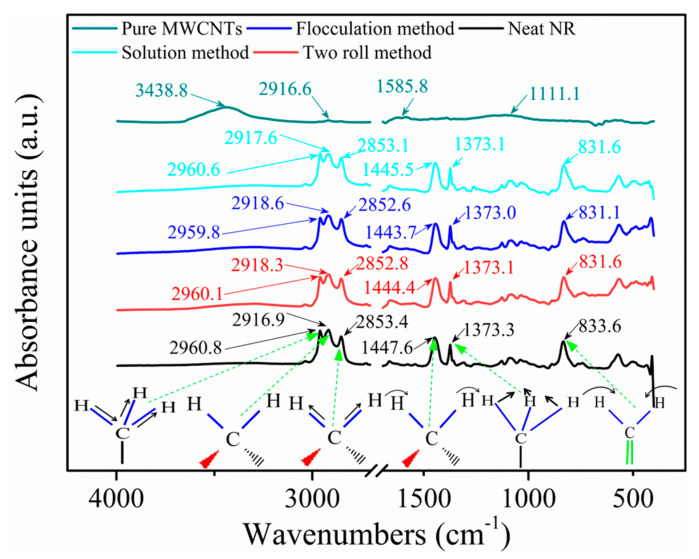
FTIR spectra of the 4 wt.% MWCNT/NR composites prepared by the different processing methods. (FTIR: Fourier transform infrared; MWCNT: multiwalled carbon nanotube; NR: natural rubber).

**Figure 13:**
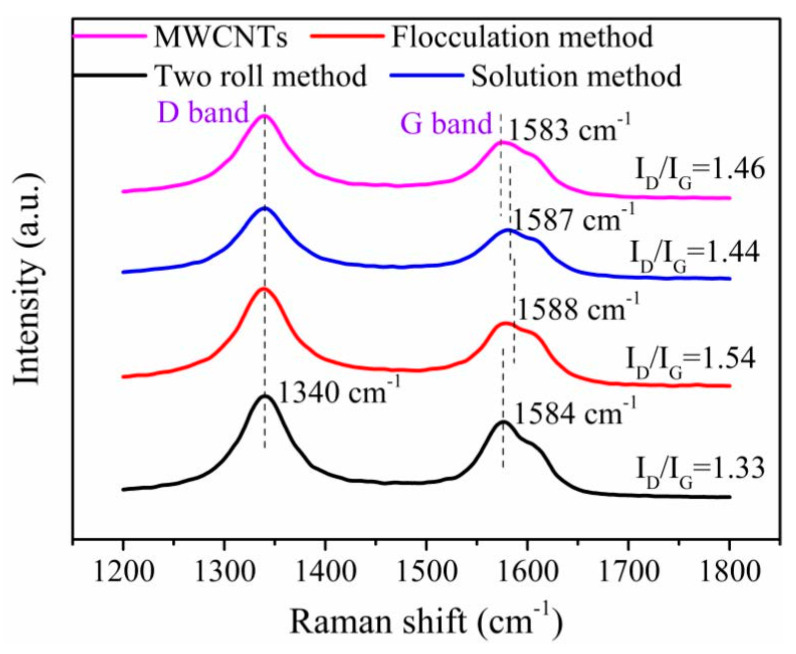
Raman spectra of the 4 wt.% MWCNT/NR composites prepared by the three preparation methods and of pure MWCNTs. (MWCNT: multiwalled carbon nanotube; NR: natural rubber; I_D_: the intensity of D band; I_G_: the intensity of G band).

Dispersion and interfacial interactions are the key factors in determining the properties of the MWCNT/NR composites. The interfacial interaction between MWCNTs and NR for the composite from the three preparation methods was investigated by FESEM, FTIR spectroscopy and Raman spectroscopy, as shown in Figure 11, Figure 12 and Figure 13.

Figure 11 shows the FESEM morphology of the composites prepared by the three methods at low magnification (red dotted) and the diameters of pull-out of MWCNTs (blue dotted). The pull-out of MWCNTs in the cross-sections and the diameters of partial MWCNTs exhibit that the three conditions have larger diameter MWCNTs than the original (Figure S1), which indicates that a polymer sheath has been produced and coated on MWCNTs [29], resulting in a larger diameter. Furthermore, the appearance of polymer sheath is closely related to the strength of interfacial interaction. In order to further explore the strength under the three processes, the following studies were carried out. The comparison finds that the flocculation method (Figure 11b) composites have rough cross-sections and fine gully-like morphologies, suggesting that good interfacial interaction between MWCNTs and NR [40,41] and good toughness [32]. However, a large number of holes can be observed in the flocculation method composite (as shown in Figure S5a), which is not good for its mechanical properties. We preliminarily believe that residual flocculant is the main factor leading to the formation of these holes (as shown in Figure S6). However, the cross-sections of the other systems show large fluctuations and morphology with a few fine gullies, especially the solution method, which may be caused by the large clusters (Figure S5b) and the structure of a segregated conductive network.

To explore the interfacial interaction of composites under different conditions, the FTIR spectroscopy was used, as shown in Figure 12. The characteristic peaks of neat NR are observed at 2960.8 cm^−1^, 2916.9 cm^−1^, 2853.4 cm^−1^, 1447.6 cm^−1^, 1373.3 cm^−1^ and 833.6 cm^−1^, corresponding to the asymmetric stretching vibration of CH_3_–, the asymmetric stretching vibration of CH_2_–, the symmetric stretching vibration of CH_2_–, the deformation of antisymmetric vibration of CH_2_–, the bending vibration of CH_3_–, and the plane bending vibration of (cis 1, 4) –CH = CH_2_ [42,43], respectively, and the vibration forms of the corresponding constituents are shown in Figure 12. The peaks of the asymmetric and symmetric stretching vibration (CH_3_–, CH_2_– and CH_2_–) for the three preparation methods shift (Figure 12), especially for the two roll method (0.7 cm^−1^, 1.4 cm^−1^, and 0.6 cm^−1^, respectively) and flocculation method (1 cm^−1^, 1.7 cm^−1^, and 0.8 cm^−1^, respectively), indicating that the interactions between the high-energy CH– of NR and MWCNTs has been developed. The two peaks corresponding to the deformation of the antisymmetric vibration of CH_2_– and the plane bending vibration of (cis 1, 4) –CH = CH_2_ of the two roll method, the flocculation method and the solution method composites show shifts of 3.2 cm^−1^, 3.9 cm^−1^; 2.1 cm^−1^, 2 cm^−1^; and 2.5 cm^−1^, 2 cm^−1^, respectively, indicating that the MWCNTs and low energy CH– have strong interaction [42]. However, composites from the three conditions exhibit changes in the four peaks of the MWCNTs: 3438.8 cm^−1^, 2916.6 cm^−1^, 1585.8 cm^−1^ and 1111.1 cm^−1^, especially at 3438.8 cm^−1^ (the stretching vibration of –OH) [44]. This result indicates that the formation of H–bonds between the MWCNTs and NR (as shown in Figure S7) is beneficial for improving the mechanical properties of the composites.

To further analyze the interfacial interaction of composites, the Raman spectra are shown in Figure 13. Clearly, the Raman spectrum of MWCNTs is observed at 1340 cm^−1^ (D band) and 1575 cm^−1^ (G band). Compared with the Raman spectrum of MWCNTs, the D band of the MWCNT/NR composites shows no obvious Raman shift, while the G band shows an obvious Raman shift of 1 cm^−1^, 5 cm^−1^ and 4 cm^−1^ for the two roll method, flocculation method and solution method composites, respectively. These results are explained by the following. The presence of NR in the composite leads to a coating of polymer on the surface of the MWCNTs, which affects the vibrations of the C–C bonds in the graphene plane due to CH–π interactions between the MWCNTs and NR [42,45], indicating that the surface of the composite is coated with NR film. The intensity ratio I_D_/I_G_ (I_D_ and I_G_ is the intensity of D band and G band, respectively) can be used to evaluate the interfacial strength of the composite [42,46]. As shown in Figure 13, the values of I_D_/I_G_ of MWCNTs, two roll method, flocculation method and solution method are 1.46, 1.33, 1.54 and 1.44, respectively. Compared with the I_D_/I_G_ values of MWCNTs, the I_D_/I_G_ values of solution and two roll methods decreased, which may be attributed to some microstructure defects within nanotubes might have been healed under the effect of shear and thermal energy, resulting in the decreasing of D band intensity and increasing of G band intensity [47]. According to the reported [43], stronger interfacial interaction contributes to higher I_D_/I_G_ value and the obvious Raman shift, which suggests that the flocculation method has a stronger interface, and the two roll method has slightly weaker interface interaction than that of the solution method. However, the existence of some holes directly limits the improvement of mechanical properties of the flocculation method. Moreover, it can well explain the change of mechanical properties of composites with MWCNT contents less than 3 wt.%.

### 3.6. Theoretical Modeling and Mechanism of the Resistance–Strain Response

As reported in the literature [34,48], the main conduction mechanism of nanocomposites with an electrical response has been dominated by tunneling or hopping between adjacent conducting particles. Thus, changes in the tunneling distance (TD) and conductive pathways (CP) play important roles in the resistance–strain response. To better understand the underlying strain sensing mechanism, a model is developed.

According to the model derived from tunneling theory [49,50], the total resistance R can be expressed using Equations (1) and (2):

(1) $$R=\left(\frac{N}{U}\right)\left(\frac{8\pi hl}{3\gamma a^{2}e^{2}}\right)\exp\left(\gamma l\right)$$

(2) $$\gamma =\frac{4\pi^{2}\sqrt{2m\xi }}{h}$$

where *N* is the number of particles forming a single conductive path, *U* is the number of conductive paths, *h* is Planck’s constant, *l* is the shortest distance between adjacent conductive particles, *a*^2^ is the effective cross-sectional area, *e* is the electron charge, *m* is the electron mass, and ξ is the height of the potential barrier between adjacent particles.

The resistance will be altered because of the separation *l* between adjacent particles when uniaxial strain is applied. The separation *l* varies linearly with the applied strain ε which can be represented as Equation (3) [51]:

(3) $$l=l_{0}\left(1+V\varepsilon \right)$$

where *l*_0_ is the initial distance between adjacent particles and *V* is a constant.

Owing to the high increasing rate of resistivity at a larger strain, it is assumed that the number of CP changes at a much higher rate, which can be expressed as follows:

(4) $$U=\frac{U_{0}}{\exp\left(\beta_{1}\varepsilon +\beta_{2}\varepsilon^{2}+\beta_{3}\varepsilon^{3}+\beta_{4}\varepsilon^{4}\right)}$$

where $\beta_{1},\;\beta_{2},\;\beta_{3}$ and $\beta_{4}$ are constants.

Substituting Equations (3) and (4) into Equation (1) yields Equation (5):

(5) $$\begin{array}{c} R=\frac{8\pi nhl_{0}}{2\gamma U_{0}{}^{2}a^{2}e^{2}}\left(1+V\varepsilon \right)\exp\left[\gamma l+\left(2\beta_{1}+\gamma lV\right)\varepsilon +2\beta_{2}\varepsilon^{2}+2\beta_{3}\varepsilon^{3}+2\beta_{4}\varepsilon^{4}\right]\\ =M\left(1+V\varepsilon \right)\exp\left[U+\left(2\beta_{1}+UV\right)\varepsilon +2\beta_{2}\varepsilon^{2}+2\beta_{3}\varepsilon^{3}+2\beta_{4}\varepsilon^{4}\right] \end{array}$$

where $M=\frac{8\pi nhl_{0}}{2\gamma U_{0}{}^{2}a^{2}e^{2}}$, U=γl.

The normalized change in resistance $\Delta R/R_{0}$ is provided by Equation (6):

(6) $$\frac{\Delta R}{R_{0}}=\frac{R}{R_{0}}-1=\left(1+V\varepsilon \right)\exp\left[\left(2\beta_{1}+UV\right)\varepsilon +2\beta_{2}\varepsilon^{2}+2\beta_{3}\varepsilon^{3}+2\beta_{4}\varepsilon^{4}\right]-1$$

**Figure 14:**
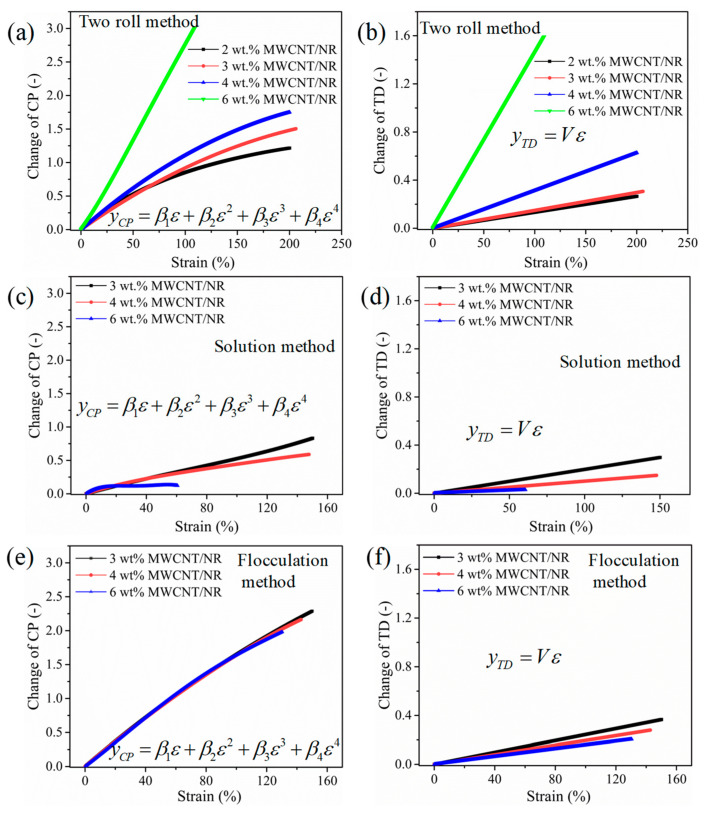
Changes in the conductive pathways and tunneling distance as a function of strain for the MWCNT/NR composites fabricated by the two roll method (**a**,**b**), solution method (**c**,**d**), and flocculation method (**e**,**f**). (MWCNT: multiwalled carbon nanotube; NR: natural rubber; CP: conductive pathway; TD: tunneling distance).

The fitting parameters and coefficients of determination (R^2^) are listed in Table 1 by fitting the $\Delta R/R_{0}$-strain curves in Figure 6 using the theoretical model (Equation (6)). The changes in CP and TD due to strain [34] are represented as Equations (7) and (8) and plotted, as shown in Figure 14, by using the parameters in Table 1.

(7) $$y_{CP}=\beta_{1}\varepsilon +\beta_{2}\varepsilon^{2}+\beta_{3}\varepsilon^{3}+\beta_{4}\varepsilon^{4}$$

(8) $$y_{TD}=V\varepsilon$$

The number of CP for the 6 wt.% MWCNT/NR composites decreases much faster than that for the 2 wt.%, 3 wt.%, and 4 wt.% MWCNT/NR composites from the two roll method and the change in TD clearly increases linearly with an increasing strain (Figure 14a,b). Moreover, the change rates of CP and TD decrease with a decreasing MWCNT content in the two roll method composites, indicating that the conductive networks undergo higher deformation under strain with an increasing MWCNT content; thus, the two roll method composites can demonstrate stronger sensitivity at a fixed strain, especially for the 6 wt.% MWCNT/NR composite. However, the opposite is true for the change rates of CP and TD for the solution (Figure 14c,d) and flocculation method (Figure 14e,f) composites. Moreover, we observe that the change rates of CP and TD for the flocculation method composite are slightly higher than those for the two roll method with 3 wt.% and 4 wt.% MWCNTs, and the conductive network density is the lowest for the 3 wt.% and 4 wt.% MWCNT/NR composites from the flocculation method. The variation in conductive network density with content and strain, as shown in Figure 14, is consistent with the experimental results in Figure 7. The result shows that the CP and TD change greatly under the same strain, thus, a higher strain sensitivity can be generated.

## 4. Conclusions

The influence of the processing method on microstructure, electrical conductivities, mechanical properties, sensitivity, dynamic sensing behaviors and interfacial interaction of MWCNT/NR composites are compared and investigated systematically. It was demonstrated that the processing method has a tremendous impact on the composite morphology and properties, some interesting conclusions are summarized as the following.

(1) Compared with the flocculation method and solution method, the two roll method can effectively reduce agglomeration and stack of MWCNTs, which is attributed to the shear stress produced by the two rolls. Meanwhile, a segregated conductive network is constructed, which is shown to be advantageous for percolation threshold (~1 wt.%) and conductivity properties when the loading is below 3 wt.%. However, the conductivity of the two roll method is lower than that of the composites prepared by the other two methods when the loading is greater than 3 wt.%.
(2) Compared with neat NR, the flocculation method shows obvious advantages in improving the mechanical properties of composites when the MWCNT contents less than 3 wt.%, but the holes and stress concentration limit the mechanical properties. Compared with the increases in conductivity for the composites obtained by the solution method and flocculation method, the composite prepared by the two roll method displays obvious improvements in its mechanical properties when the MWCNT content is higher than 3 wt.%, due to the formation of the segregated networks even if the interface interaction is weak.
(3) The resistance–strain response behavior of the composite prepared by the two roll method shows high sensitivity (*GF*_max_ = 974.2) and a wide monitoring range (*ε* = 109%). Meanwhile, the elimination of the ‘shoulder peak’ and better stability and repeatability of the resistance–strain response are achieved by the two roll method when compared with the other two methods. The solution method exhibits excellent *GF* stability during strain cycles, but the extremely small *GF* and strong ‘shoulder peak’ will be the key to limit its application.
(4) The mechanism of the resistance–strain response is investigated by employing an analytical model. The comparisons of the fractional resistance change between the measured results and the theoretical model indicate that the employed model can characterize and explain the resistance–strain response quite well.

## Figures and Tables

**Table 1:** Parameters obtained by fitting the ∆*R*/*R*_0_–strain curves in Figure 7. (∆*R*/*R*_0_: the resistance change fraction).

Preparationd	Composite	*U*	*V*	*β*_1_	*β*_2_	*β*_3_	*β*_4_	*R* ^2^
Two roll method	2 wt.% MWCNT/NR	3.8373	0.1328	1.3330	−0.6571	0.2069	−0.0298	1.0
	3 wt.% MWCNT/NR	1.8515	0.1488	1.1010	−0.1809	−0.0010	0.0007	0.999
	4 wt.% MWCNT/NR	3.0880	0.3137	1.3790	−0.2981	0.0352	−0.0060	1.0
	6 wt.% MWCNT/NR	0.000035	1.4580	2.0590	1.9130	−1.7910	0.5694	1.0
Solution method	3 wt.% MWCNT/NR	1.6111	0.2551	1.9650	−0.2781	0.0185	−0.0036	0.998
	4 wt.% MWCNT/NR	8.5532	0.1963	1.8970	−0.3147	0.1068	−0.0514	0.999
	6 wt.% MWCNT/NR	1.0362	0.1575	1.6960	0.5864	−0.9421	0.2983	0.998
Flocculation method	3 wt.% MWCNT/NR	0.4849	0.1983	0.6269	−0.1919	0.0826	0.0086	1.0
	4 wt.% MWCNT/NR	0.9921	0.1008	0.7468	−0.5868	0.3785	−0.0953	1.0
	6 wt.% MWCNT/NR	13.031	0.0466	1.4920	−7.2110	14.610	−10.280	0.999

## Data Availability

The data presented in this study are available on request from the corresponding author.

## Supplementary Materials

The following are available online at https://www.mdpi.com/article/10.3390/nano11071845/s1, Figure S1: The diameter and micromorphology of original multiwalled carbon nanotubes are characterized by transmission electron microscopy, Figure S2: XRD spectrum of (a) pure MWCNTs, (b) 6 wt.% MWCNT/NR and (c) 6 wt.% CTAB/NR of the flocculation method, Figure S3: Mechanical properties: (a) tensile strength, (b) modulus at 100% strain, and (c) elongation at break of the MWCNT/NR composites prepared by the three processing, Figure S4: Ln(Gauge factor) of 6 wt.% MWCNT/NR prepared by solution method as function of applied strain, Figure S5: The cross-section micro-topography of 6 wt.% MWCNT/NR composites under (a) flocculation method and (b) solution method, Figure S6: Micromorphology of CTAB/MWCNT/NR composite without flocculant, Figure S7: Schematic diagram of H-bonding interactions between MWCNTs and NR, Table S1: Review of nanocomposite strain sensor literatures.
