# Supplementary material for: Effect of the Processing on the Resistance–Strain Response of Multiwalled Carbon Nanotube/Natural Rubber Composites for Use in Large Deformation Sensors
Source: Nanomaterials (Basel). 2021 Jul 16;11(7):1845. doi: 10.3390/nano11071845 (PMC8308229; doi:10.3390/nano11071845)
Supplement: Supplementary file 1 [file nanomaterials-11-01845-s001.zip › nanomaterials-1281697-supplementary.pdf]

nanomaterials

MDPI

Article

Effect of the Processing on the Resistance–Strain Response of Multiwalled Carbon Nanotube/Natural Rubber Composites for Use in Large Deformation Sensors

Xingyao Liu, Rongxin Guo *, Rui Li, Hui Liu, Zhengming Fan, Yang Yang and Zhiwei Lin

Key Laboratory of Disaster Reduction in Civil Engineering, Faculty of Civil Engineering and Mechanics, Kunming University of Science and Technology, Kunming 650500, China; xingyaoliu@stu.kust.edu.cn (X.L.); liruiking@kust.edu.cn (R.L.); 15079743713@163.com (H.L.); 20202210032@stu.kust.edu.cn (Z.F.); yangyang0416@kust.edu.cn (Y.Y.); lzw@kust.edu.cn (Z.L.)

* Correspondence: guorx@kmust.edu.cn

1. Characterizations

1.1. Field Emission Scanning Electron Microscopy (FESEM)

FESEM (Zeiss S4800, Carl Zeiss AG, Oberkochen, Germany) was used to characterize the fracture morphology of Multiwalled Carbon Nanotube (MWCNT)/ Natural Rubber (NR) and the dispersion of MWCNT in NR at an accelerated voltage of 10 kV. Samples were frozen in liquid nitrogen for 5 min, and then bending forces were applied to break samples to obtain new fracture surfaces. Gold-sprayed treatment was carried out on the fracture surface of the sample to improve the conductivity and obtain better FESEM images. In this work, composite samples prepared by different processing method were tested.

1.2. Transmission Electron Microscopy (TEM)

The dispersion and exfoliation of MWCNTs in NR were characterized by TEM (FEI Talos F200s, Thermo Fisher Scientific, MA, USA). The samples of TEM with a thickness of 80 nm were prepared under freezing conditions by Leica UC7 instrument and then collected on a copper network.

1.3. X-Ray Diffraction (XRD)

XRD was used to characterize the aggregation and stacking of MWCNTs in NR. Spectra were performed under Cu Ka radiation and a reflection mode at 2°/min between 5° and 90°.

1.4. Fourier Transform Infrared (FTIR)

Attenuated total reflection Fourier transform infrared spectroscopy（ATR-FTIR）(Thermo Scientific IS5, Thermo Fisher Scientific, MA, USA) used to prove the interfacial interaction between MWCNTs and NR in this work, the analysis was carried out at the resolution of 4 cm^{-1} and the wavenumber range of 4000–400 cm^{-1}.

1.5. Raman Spectroscopy

Raman spectroscopy (HORIBA Scientific LabRAM HR Evolution, HORIBA Jobin Yvon, Paris, France) was used to analyze the interface with a Raman shift of 800–2000 cm^{-1}.

1.6. Stress-Strain

The stress-strain characteristics of the dumb bell standard specimens (according to ISO 37:2005(E)) were measured by a tensile and compression test system (DDL-10) with a maximum load of 10 kN controlled by displacement at ambient temperature, and the loading rate was 200 ± 5 mm/min according to ISO 37:2005(E). The number of samples in each experiment was no less than three.

1.7. Conductivity

To measure the electrical conductivity of the MWCNT/NR composites, the electrical signals (all sizes are 40 mm × 10 mm × 1 mm) were measured with a Keysight 34410A device (Keysight Technologies, Inc., CA, USA). The two-wire method was used to directly measure the resistance of the samples under all conditions. To ensure good contact between the crocodile-snout clamp and the sample, copper sheet and conductive silver glue are used.

According to the resistance value, the volume conductivity of samples was calculated by combining Equations S(1)–S(3).

The resistance and conductivity are directly related to the geometry of samples:

$$R = \rho \frac{t}{A} \tag{S1}$$

where R is the resistance of the samples, t is the thickness, A is the cross-sectional area and ρ represents the resistivity. The conductance G has the following relation with resistance:

$$G = \frac{1}{R} \tag{S2}$$

Conductivity is defined as the current flowing through 1 cm^3 of the material per unit potential. The relationship between the conductivity and conductance is shown below:

$$\sigma = \frac{1}{\rho} = G\frac{t}{A} = \frac{t}{RA} \tag{S3}$$

where σ represents the volume conductivity.

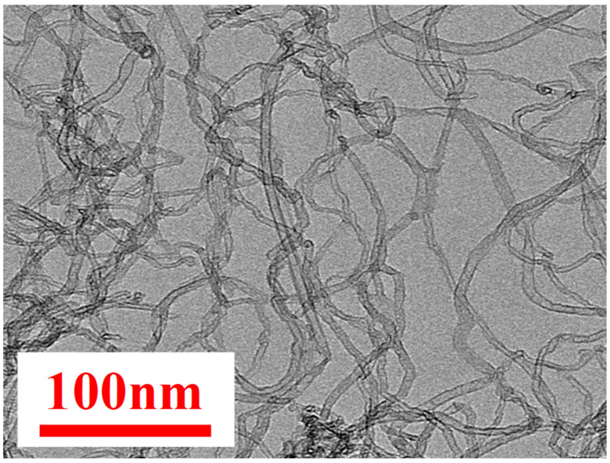

Figure S1. The diameter and micromorphology of original multiwalled carbon nanotubes are characterized by transmission electron microscopy. (Note: the data of Figure S1 is provided by the *Chengdu Organic Chemicals Co., Ltd., Chinese Academy of Sciences, China*).

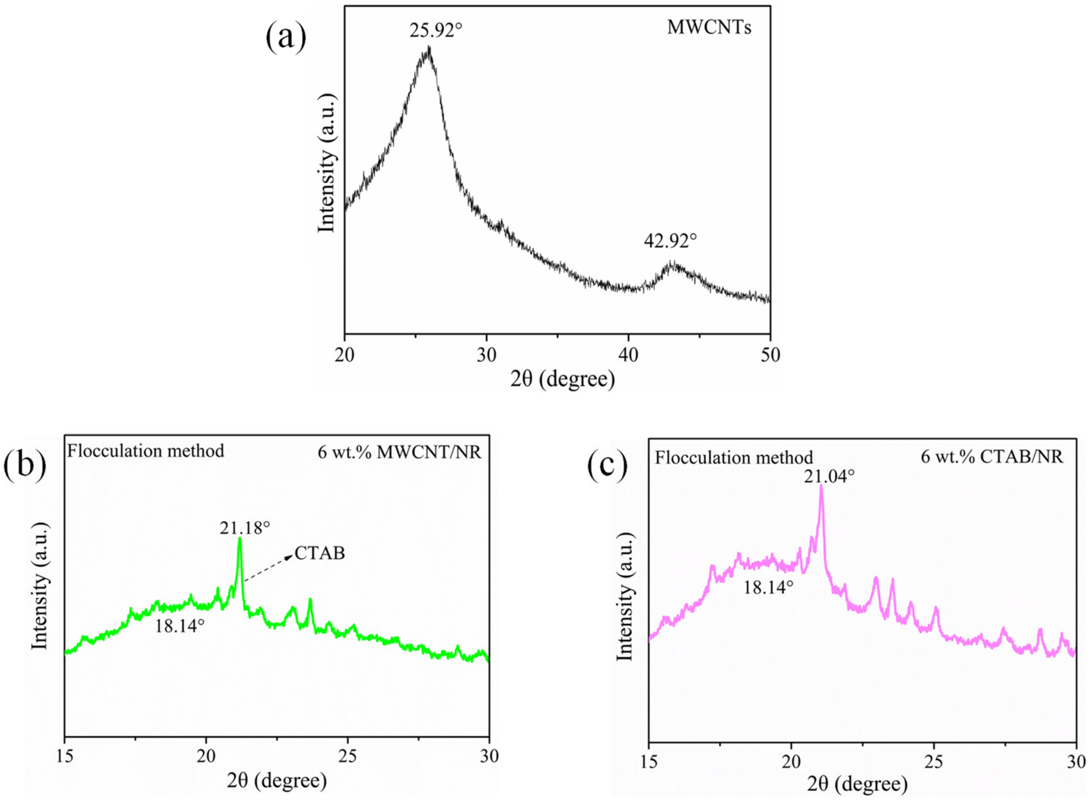

Figure S2. XRD spectrum of (**a**) pure MWCNTs, (**b**) 6 wt.% MWCNT/NR and (**c**) 6 wt.% CTAB/NR of the flocculation method. (XRD: X-ray diffraction; MWCNT: multiwalled carbon nanotube; NR: natural rubber; CTAB: cetyl trimethyl ammonium bromide).

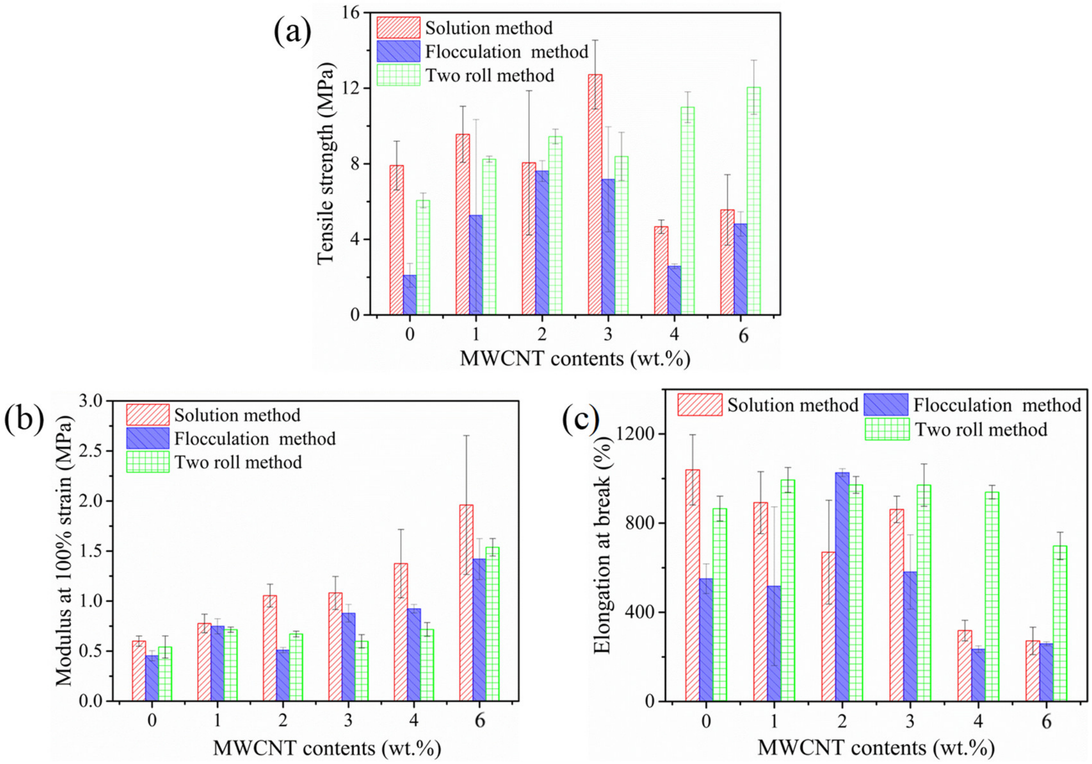

Figure S3. Mechanical properties: (**a**) tensile strength, (**b**) modulus at 100% strain, and (**c**) elongation at break of the MWCNT/NR composites prepared by the three processing. (MWCNT: multiwalled carbon nanotube; NR: natural rubber).

Regarding both the solution method and flocculation method, it is clear that the tensile strength (TS) of the MWCNT/NR composites significantly increases when the MWCNT content increases at low loading (Figure S3a); these TS reach maximum values at 3 wt.% and 2 wt.% MWCNTs, respectively. And then the TS of these composites decrease when the MWCNT content continues to increase due to the stress concentration caused by a high filler content. However, it is surprising to note that the TS of composite prepared by the two roll method increases almost linearly with the MWCNT content (Figure S3a). For instance, the TS of 6 wt.% MWCNT/NR composite prepared by the two roll method is 2.5 times and 2.1 times higher than that of composites from the flocculation method and solution method, respectively; additionally, the TS is increased by 99% when compared with neat NR, indicating that the two roll method does not cause severe filler aggregation and that the stress concentration is weak. Regarding the two roll method, the increase in moduli at 100% strain (M100) (Figure S3b) is slow at first and then increases rapidly. In contrast, the other two methods demonstrate a stable increase with an increasing filler content. Furthermore, the elongation at break for the composite from the solution method decreases gradually with an increasing filler content, as shown in Figure S3c. Regarding the other two systems, the elongation at break first increases and then decreases gradually with the filler content, and reached the highest value at their respective thresholds. At a fixed filler loading of 6 wt.%, the elongation at break of the two roll method merely decreases by 24% with respect to NR and is 2.7 times and 2.6 times that of the solution method and flocculation method, respectively.

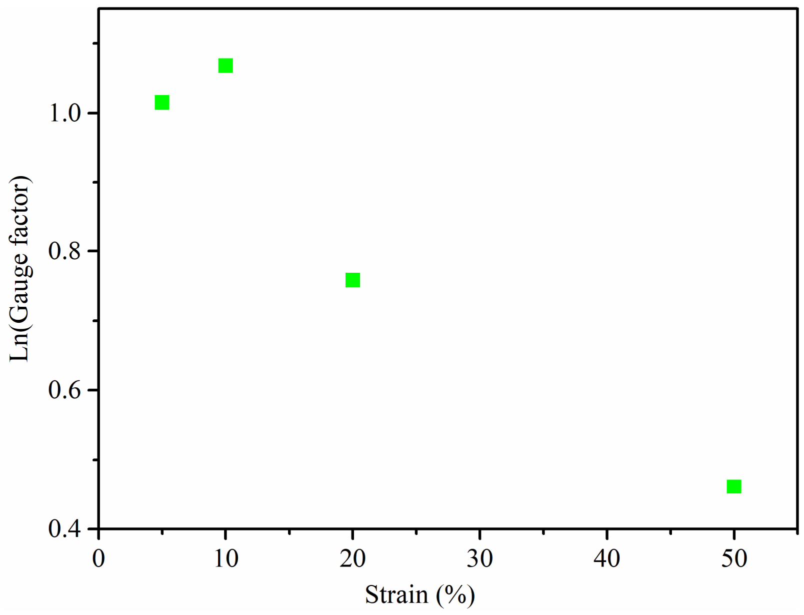

Figure S4. Ln (Gauge factor) of 6 wt.% MWCNT/NR prepared by solution method as function of applied strain. (MWCNT: multiwalled carbon nanotube; NR: natural rubber).

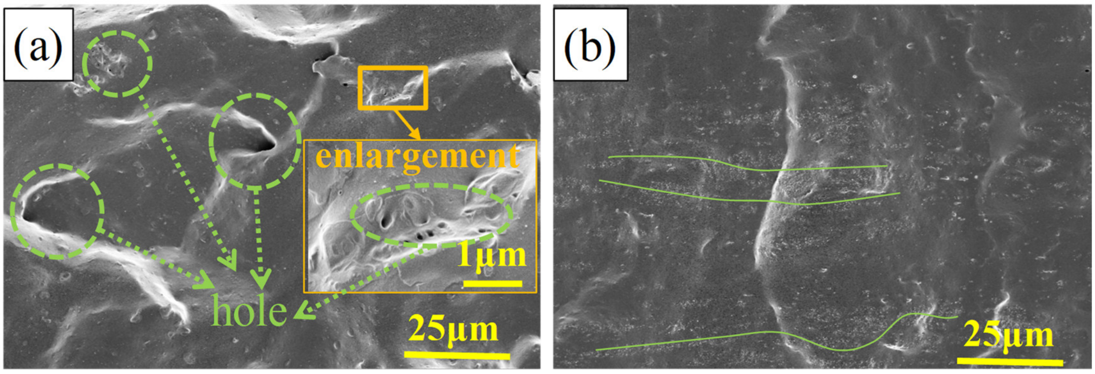

Figure S5. The cross-section micro-topography of 6 wt.% MWCNT/NR composites under (**a**) flocculation method and (**b**) solution method. (MWCNT: multiwalled carbon nanotube; NR: natural rubber).

Figure S5 is the FESEM morphology of the composites prepared by flocculation and solution method. Figure S5a shows that there are many and dense holes in the flocculation composites, as shown by the dotted green circle and the inset, and the existence of these holes affects the mechanical properties of the composites. Figure S5b shows the presence of a large number of MWCNT agglomeration bands in the solution method composites, as shown by the green line in the Figure S5b.

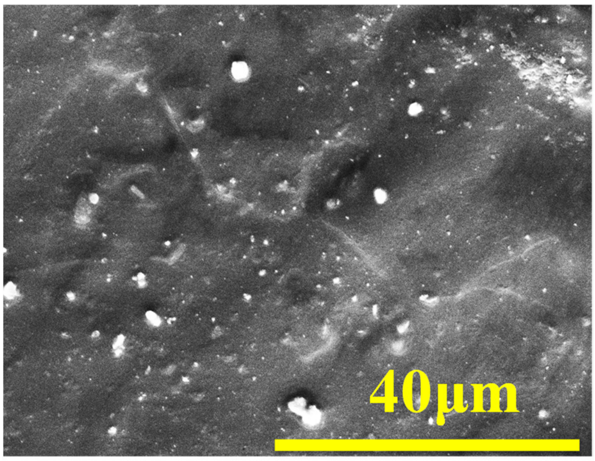

Figure S6. Micromorphology of CTAB/MWCNT/NR composite without flocculant. (CTAB: cetyl trimethyl ammonium bromide; MWCNT: multiwalled carbon nanotube; NR: natural rubber).

In order to verify that the large number of holes in the flocculating method in the main document are related to the existence of flocculant, we carried out microscopic morphology experiment on the composite without flocculant, as shown in the Figure S6, which shows that holes not appear on the micromorphology of the cetyl trimethyl ammonium bromide (CTAB)/MWCNT/NR composite without flocculant, indicating that the existence of holes in Figure S5(a) should be related to flocculant.

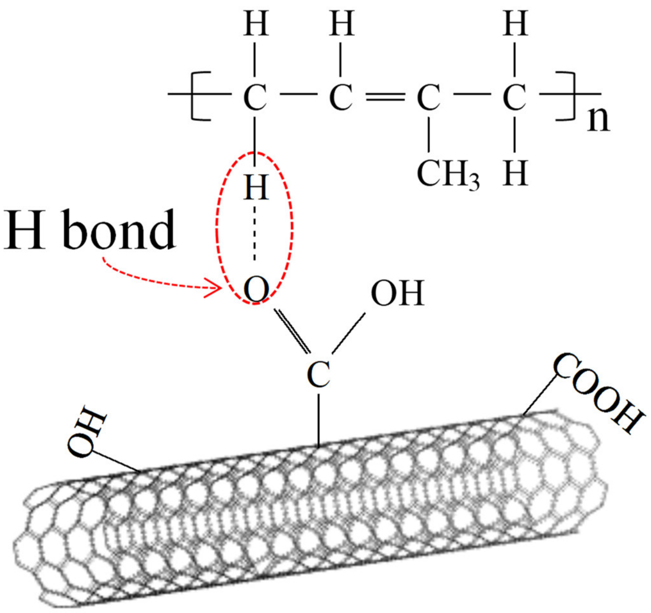

Figure S7. Schematic diagram of H-bonding interactions between MWCNTs and NR. In the figure, the diagram of single-walled carbon nanotube is used instead of multi walled carbon nanotube, which does not affect the meaning of the figure. (MWCNT: multiwalled carbon nanotube; NR: natural rubber).

Table S1. Review of nanocomposite strain sensor literatures.

Reference Number	Nanofiller Content	Strain (%)	Gauge Factor	Materials
[1]	0.2 vol.	667	35	Graphene/Natural rubber
[2]	35 vol.	80	20	Carbon Black/Silicone
[3]	N/A	280	0.82	SWCNT/PDMS
[4]	0.21 vol.	20	2.6	MWCNT/vinyl ester
[5]	0.143 vol.	403	4	MWCNT/PU
[6]	0.63 vol.	90	6.9	Graphene/Natural rubber
[7]	0.011vol.	30	5.4	Graphene/TPU
[8]	2.45 vol.	30	4.36	CNTs/PDMS
[9]	15 wt.	80	5.5	Carbon Black/ PDMS
[10]	N/A	150	1.4	Graphene/Nylon covered rubber
[11]	4.0 wt.	135	6.8	CNTs/TPU
[12]	4.5 vol.	220	5.4	CNTs/poly(m-phenylene isophthalamide)
[13]	1.0 wt.	45	7	MWCNT/TPU
[14]	6.0 wt.	20	8	CNTs/CB/SEBS
[15]	0.9 vol.	160	149	Graphene/Natural rubber
**This work**	**6 wt.**	**109**	**974.2**	**MWCNT/Natural rubber**

References

1. Boland, C.S.; Khan, U.; Backes, C.; O'Neill, A.; McCauley, J.; Duane, S.; Shanker, R.; Liu, Y.; Jurewicz, I.; Dalton, A.; et al. Sensitive, High-Strain, High-Rate Bodily Motion Sensors Based on Graphene–Rubber Composites. *ACS Nano* **2014**, *8*, 8819–8830, doi:10.1021/nn503454h.
2. Mattmann, C.; Clemens, F.; Tröster, G. Sensor for Measuring Strain in Textile. *Sensors* **2008**, *8*, 3719–3732, doi:10.3390/s8063719.
3. Yamada, T.; Hayamizu, Y.; Yamamoto, Y.; Yomogida, Y.; Izadi-Najafabadi, A.; Futaba, D.; Hata, K. A stretchable carbon nanotube strain sensor for human-motion detection. *Nat. Nanotechnol.* **2011**, *6*, 296–301, doi:10.1038/nnano.2011.36.
4. Ku-Herrera, J.J.; Avilés, F. Cyclic tension and compression piezoresistivity of carbon nanotube/vinyl ester composites in the elastic and plastic regimes, *Carbon* **2012**, *50*, 2592–2598.
5. Slobodian, P.; Riha, P.; Saha, P. A highly-deformable composite composed of an entangled network of electrically-conductive carbon-nanotubes embedded in elastic polyurethane. *Carbon* **2012**, *50*, 3446–3453, doi:10.1016/j.carbon.2012.03.008.
6. Lin, Y.; Dong, X.; Liu, S.; Chen, S.; Wei, Y.; Liu, L. Graphene–Elastomer Composites with Segregated Nanostructured Network for Liquid and Strain Sensing Application. *ACS Appl. Mater. Interfaces* **2016**, *8*, 24143–24151, doi:10.1021/acsami.6b08587.
7. Liu, H.; Li, Y.; Dai, K.; Zheng, G.; Liu, C.; Shen, C.; Yan, X.; Guo, J.; Guo, Z. Electrically conductive thermoplastic elastomer nanocomposites at ultralow graphene loading levels for strain sensor applications. *J. Mater. Chem. C* **2016**, *4*, 157–166, doi:10.1039/c5tc02751a.
8. Zheng, Y.; Li, Y.; Li, Z.; Wang, Y.; Dai, K.; Zheng, G.; Liu, C.; Shen, C. The effect of filler dimensionality on the electromechanical performance of polydimethylsiloxane based conductive nanocomposites for flexible strain sensors. *Compos. Sci. Technol.* **2017**, *139*, 64–73, doi:10.1016/j.compscitech.2016.12.014.
9. Kong, J.-H.; Jang, N.-S.; Kim, S.-H.; Kim, J.-M. Simple and rapid micropatterning of conductive carbon composites and its application to elastic strain sensors. *Carbon* **2014**, *77*, 199–207, doi:10.1016/j.carbon.2014.05.022.
10. Park, J.J.; Hyun, W.J.; Mun, S.C.; Park, Y.T.; Park, O.O. Highly Stretchable and Wearable Graphene Strain Sensors with Controllable Sensitivity for Human Motion Monitoring. *ACS Appl. Mater. Interfaces* **2015**, *7*, 6317–6324, doi:10.1021/acsami.5b00695.
11. Zheng, Y.; Li, Y.; Dai, K.; Liu, M.; Zhou, K.; Zheng, G.; Liu, C.; Shen, C. Conductive thermoplastic polyurethane composites with tunable piezoresistivity by modulating the filler dimensionality for flexible strain sensors. *Compos. Part A: Appl. Sci. Manuf.* **2017**, *101*, 41–49, doi:10.1016/j.compositesa.2017.06.003.
12. Jiang, S.; Zhang, H.; Song, S.; Ma, Y.; Li, J.; Lee, G.H.; Han, Q.; Liu, J. Highly Stretchable Conductive Fibers from Few-Walled Carbon Nanotubes Coated on Poly(m-phenylene isophthalamide) Polymer Core/Shell Structures. *ACS Nano* **2015**, *9*, 10252–10257, doi:10.1021/acsnano.5b04185.
13. Kumar, S.; Gupta, T.K.; Varadarajan, K. Strong, stretchable and ultrasensitive MWCNT/TPU nanocomposites for piezoresistive strain sensing. *Compos. Part B: Eng.* **2019**, *177*, 107285, doi:10.1016/j.compositesb.2019.107285.
14. Yang, X.; Sun, L.; Zhang, C.; Huang, B.; Chu, Y.; Zhan, B. Modulating the sensing behaviors of poly(styrene-ethylene-butylene-styrene)/carbon nanotubes with low-dimensional fillers for large deformation sensors. *Compos. Part B: Eng.* **2019**, *160*, 605–614, doi:10.1016/j.compositesb.2018.12.119.
15. Yang, H.; Yao, X.; Zheng, Z.; Gong, L.; Yuan, L.; Yuan, Y.; Liu, Y. Highly sensitive and stretchable graphene-silicone rubber composites for strain sensing. *Compos. Sci. Technol.* **2018**, *167*, 371–378, doi:10.1016/j.compscitech.2018.08.022.
